# Molecular investigation of *Dirofilaria repens*, *Dirofilaria immitis* and *Acanthocheilonema reconditum* in stray dogs and cats in Ukraine

**DOI:** 10.1186/s12917-025-04867-w

**Published:** 2025-07-05

**Authors:** Mateusz Pękacz, Kateryna Slivinska, Alla Vyniarska, Katarzyna Basałaj, Alicja Kalinowska, Agnieszka Wesołowska, Alicja Laskowska, Olesia Kysterna, Andrii Klietsov, Martina Miterpáková, Andrei Daniel Mihalca, Jakub Gawor, Vitaliy Kharchenko, Anna Zawistowska-Deniziak

**Affiliations:** 1 Department of Immunology, Institute of Experimental Zoology, Faculty of Biology, University of Warsaw, Warsaw, 02-096 Poland; 2 Museum and Institute of Zoology, Polish Academy of Sciences, Warsaw, 00-679 Poland; 3 I.I. Schmalhausen Institute of Zoology of National Academy of Sciences of Ukraine, Kyiv, 01030 Ukraine; 4 Stepan Gzhytskyi National University of Veterinary Medicine and Biotechnologies of Lviv, Lviv, 79010 Ukraine; 5 Department of Therapy, Pharmacology, Clinical Diagnostics and Biochemistry, Sumy National Agrarian University, Sumy, 40021 Ukraine; 6 NGO “Society of Veterinary Business Owners”, Sumy, Ukraine; 7 Institute of Parasitology, Slovak Academy of Sciences, Košice, 04001 Slovakia; 8 Department of Parasitology and Parasitic Diseases, University of Agricultural Sciences and Veterinary Medicine of Cluj-Napoca, Cluj-Napoca, 400372 Romania; 9 Polish Advisory Council for Parasitoses of Companion Animals ESCCAP Poland, Warsaw, 02-001 Poland; 10 Faculty of Biology, Genomics and Transcriptomics Laboratory, University of Warsaw, Warsaw, 02-096 Poland

**Keywords:** *Acanthocheilonema*, *Dirofilaria*, Molecular epidemiology

## Abstract

**Background:**

The increasing population of stray dogs and cats in Ukraine poses an important risk of the transmission of vector-borne parasites, particularly *Dirofilariarepens*, *Dirofilaria immitis* and *Acanthocheilonema reconditum*, all of which are zoonotic and may affect human health. Despite numerous reports of human dirofilariosis in Ukraine, epidemiological data on these filarial parasites in companion animals remain limited. The aim of the study was to conduct a molecular epidemiological survey to assess the prevalence of filarial infections in stray dogs and cats across Ukraine and evaluate factors associated with infection in dogs. In collaboration with the European Scientific Counsel Companion Animal Parasites (ESCCAP) and local non-governmental organizations (NGOs), a total of 457 blood samples (233 dogs and 224 cats) were collected between March and December 2023 from Berdychiv, Lviv, Kharkiv, Sumy and Zvenyhorodka. Molecular detection of *D. repens*, *D. immitis*, and *A. reconditum* was performed using a two-step quantitative PCR (qPCR) assay with novel species-specific primers.

**Results:**

The method demonstrated high sensitivity and specificity, capable of detecting DNA from a single microfilaria, with no evidence of cross-reactivity among target species. Among the canine samples, 66 (28.3%) tested positive for at least one filarial species, including cases of both mono- and co-infection. In contrast, only 8 feline samples (3.6%) were positive for *D. repens* or *D. immitis*. Statistical analysis revealed a higher prevalence among male dogs and those weighing over 10 kg, while the lowest prevalence was observed in the youngest age group (< 3 years).

**Conclusions:**

These findings provide the first molecular evidence of *Dirofilaria* spp. and *A. reconditum* infections in stray animals from several under-studied areas of Ukraine, highlighting the relevance of the One Health approach in mitigating the risk of zoonotic transmission.

**Supplementary Information:**

The online version contains supplementary material available at 10.1186/s12917-025-04867-w.

## Background

Dirofilariosis is a vector-borne disease, caused by parasitic nematodes transmitted by mosquitoes, that is rapidly spreading across the Old World. While dogs are the predominant reservoirs, an increasing variety of domestic and wild species are now considered at risk [1–4]. The pathogens in question, *Dirofilaria repens* and *Dirofilaria immitis*, are responsible for subcutaneous and cardiopulmonary dirofilariosis, respectively, and are prevalent across Europe to varying degrees [5, 6]. Recent research indicates evolving prevalence patterns of *Dirofilaria* spp., including their detection in previously unaffected areas [6, 7], and, in some instances, a shift in dominance between species [8, 9].

Given that both pathogens are endemic in Ukraine,, the epidemiological study of canine and feline dirofilariosis remains limited [10–12]. Paradoxically, this country has recorded the highest number of human *Dirofilaria* infecions worldwide. Ukraine is likely the only European nation with a mandatory case registration system, along with a national register for *Dirofilaria* human infections, in place since 1975. Between 1997 and 2012 alone, 1,465 cases, primarily caused by *D. repens*, were noted [13], with a limited number of cases were also attributed to *D. immitis* [14]. In contrast, between 2000 and 2019, only 576 clinical cases were reported worldwide outside of Ukraine [15], underscoring the important burden of dirofilariosis in the country. Despite climate change driving an increase in vector-borne diseases [16], the lack of knowledge regarding canine dirofilariosis in the area further complicates the situation. The transmission of dirofilariosis is also heavily influenced by the growing population of stray animals. Prior to 2022, the number of stray dogs in Ukraine was estimated at approximately 200,000 dogs, with an even larger population of free-roaming cats [17]. The situation has deteriorated dramatically following Russia’s military invasion, exacerbating challenges in managing the stray animal population. According to the UNHCR’s United Nations High Commissioner for Refugees’ (UNHCR) January 2024 report, over 6.3 million people have fled Ukraine, and 3.7 million have been displaced within the country [18]. As a result, monitoring and controlling the stray animal population has become increasingly difficult. Without adequate care and preventative measures, they can become primary carriers of a wide spectrum of diseases, including parasitic infections. The possibility of both common and rare diseases spreading to traditionally non-endemic areas is heightened by the animals’ unregulated breeding. This situation facilitates the persistence of vector-borne parasitic infections, such as dirofilariosis, by sustaining parasite transmission cycles in the environment and maintaining a continuous risk of infection for both animals and humans. In this context, and within the collaborative, interdisciplinary One Health framework, the present study aimed to: (1) update the prevalence data of *Dirofilaria* spp. and *A. reconditum* in dogs, and for the first time, in cats from Ukraine; and (2) analyze factors potentially influencing filarial infections in dogs, including sex, weight, and age.

## Methods

### Sample collection and preservation

Conducted in partnership with European Scientific Counsel Companion Animal Parasites (ESCCAP) and several Ukrainian non-governmental organizations (NGO), this research integrates parasitological examinations into a broader national initiative for the sterilization and vaccination of stray animals. Blood samples were collected from randomly caught stray dogs and cats in regions where the programme was being implemented. Specimens were drawn into EDTA tubes during procedures performed by NGO-affiliated veterinarians. Following these interventions, the animals were transferred to local shelters or new caregivers. Post-collection, the samples were refrigerated at 4 °C until further analysis.

### Sample demographics and geographical distribution

**Fig. 1:**
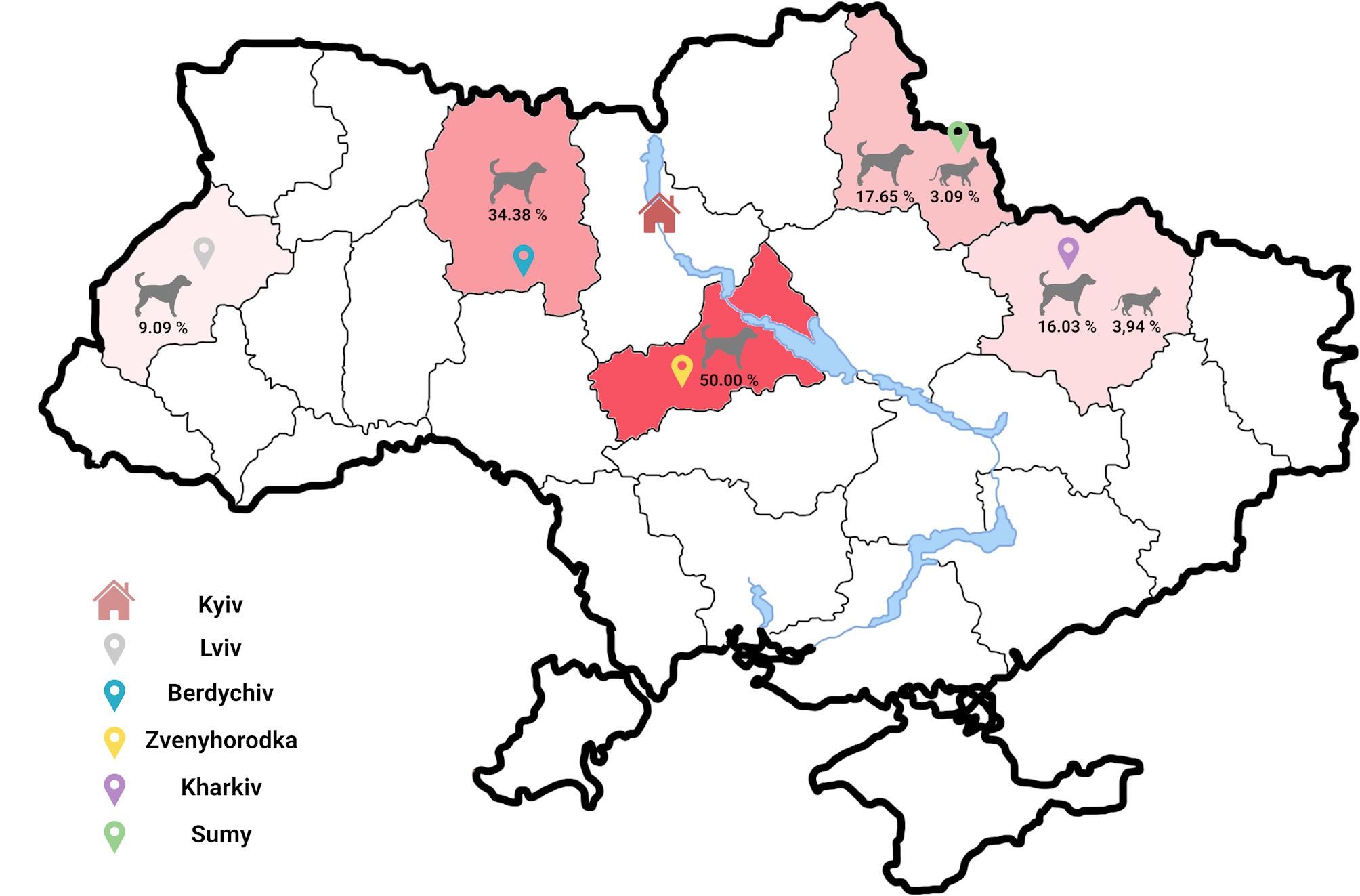
The geographic distribution of filariae infections in Ukrainian stray dogs and cats. The administrative areas where the investigation was conducted were labeled in varying shades of red, reflecting the frequency of occurrence. The cities where the investigation was conducted were marked with pins of different colors: grey for Lviv, blue for Berdychiv, yellow for Zvenyhorodka, purple for Kharkiv, and green for Sumy. The capital city of Ukraine, Kyiv, was indicated by a house icon

A total of 465 blood samples were obtained from stray dogs (*n* = 233) and cats (*n* = 224) populations between March and December 2023. The collection encompassed five urban locations within Ukraine: Berdychiv (49°54′0″N 28°34′0″E, Lviv (49°50′33″N 24°01′56″E), Kharkiv (49°59′33″N 36°13′52″E), Sumy (50°54′43″N 34°48′10″E) and Zvenyhorodka (49°4′11″N 30°58′4″E) as illustrated in Fig. 1. A detailed enumeration of the sample distribution across these areas is delineated in Additional File 1: Table S1.

### Genomic DNA extraction and Real-Time PCR assay

Genomic DNA (gDNA) was extracted from canine and feline blood samples, using 300 µl and 100 µl volumes, respectively, with the Blood Mini Kit (AA Biotechnology), following the supplier’s instructions. The extracted gDNA served as the template for the subsequent Real-Time PCR assay aimed at identifying *Dirofilaria* spp. Initially, each sample was tested with the internal extraction/amplification controls, using primers targeting the *LINE-1* gene in dogs [19] and the *28 S rRNA* gene in cats [20] (Additional File 1: Fig. S1). Following this, samples were analyzed with primers that amplify a conserved fragment of the *cox1* gene, common within the family Onchocercidae family. Positive samples then underwent species-specific amplification to identify *D. repens*, *D. immitis*, and *A. reconditum*, using species-specific primers (Table 1). Both the pan-filarial and species-specific primers were newly designed for this study.

**Table 1:** The list of primers used in Real-Time PCR for internal extraction and amplification control, as well as for the detection and differentiation of filarial infections

Primer name	Sequence (5’ -> 3’)	Gene	Family/species	Amplicon size	Reference
For_LINE1_dog	CAAATGCAATGAAACGCCGGGACA	*LINE1*	*Canis lupus familaris*	100 bp	[19]
Rev_LINE1_dog	TCTTTCGTTGGACACCGAGGCTC				
For_28s_cat	CGCTAATAGGGAATGTGAGCTAG	*28 S rRNA*	*Felis catus*	121 bp	[20]
Rev_28s_cat	TGTCTGAACCTCCAGTTTCTCTG				
For_cox1_filariae	GGGTAATCCTTTGTTGTATCAGCATTTG	*cox1*	Onchocercidae	133 bp	This study
Rev_cox1_filariae	GCCAAACAAACGATCCTTATCAGTCAA				
For_s16_Dr	CTCCGGAGTTAACAGGGTTGTAGA	*16 S rRNA*	*D. repens*	116 bp	This study
Rev_s16_Dr	CAGTCTCAAAAAAAAAACAATCTCTCCTCC				
For_CytB_Di	CTATTCTTATTTGACCGGGTGCG	*cytB*	*D. immitis*	123 bp	This study
Rev_CytB_Di	GATAATCAGTAGGATAATACCCAGCT				
For_ITS_Ar	GTCAGGTGATGGTTTGATGTGC	*ITS*	*A. reconditum*	84 bp	This study
Rev_ITS_Ar	ATTGTGTGCCAACTGTATACTGCT				

Each reaction was performed in triplicate for the target gene, incorporating a melting curve analysis, utilizing the QuantStudio 6 Real-Time PCR system (Applied Biosystems) in alignment with the PowerUp SYBR Green Master Mix fast cycling protocol (Applied Biosystems). The PCR mix included 3 µl of gDNA, 5 µl of PowerUp SYBR Green Master Mix (2×), both primers at a 0.6 µM final concentration, and nuclease-free water to a total volume of 10 µl. The specificity of PCR products was ascertained through melt curve analysis alongside known references, supplemented by Sanger sequencing for definitive confirmation. Sequencing of the amplicons was outsourced to Genomed S.A., where bidirectional sequencing was performed using the same gene-specific primers as in qPCR assay.

### Evaluation of analytical sensitivity and specificity of the qPCR assay with newly designed primers

To evaluate the sensitivity of the qPCR assay with the use of newly designed primers, single microfilariae were obtained from blood of a dog infected with *D. repens*. One, three, or 10 live microfilariae were isolated under the microscope and transferred into fresh tubes containing 300 µL of fresh blood from an uninfected dog. Genomic DNA (gDNA) was then extracted as described in the manuscript, eluted with the same volume (150 µL), and 3 µL was used for qPCR with primers targeted at *cox1*. Each solution (1 mf, 3 mf, 10 mf) was repeated in three biological groups, using blood from three distinct healthy dogs. Blood samples from both *Dirofilaria*-positive and -negative dogs were obtained as leftovers from diagnostic procedures performed by veterinarians at clinics in Warsaw, Poland. Sensitivity of the qPCR assay was tested using pan-filarial primers targeting *cox1* (For_cox1_filariae; Rev_cox1_filariae), as this reaction is critical for diagnosis.

The specificity of the newly designed primers used in the qPCR assay was evaluated: pan-filarial primers were tested for their ability to amplify multiple filarial species, while species-specific primers aimed to prevent cross-priming with non-target filarial species. Reference specimens for *D. repens* and *D. immitis* were obtained from prior investigations [21]. Additionally, gDNA from dogs concurrently infected with both *Dirofilaria* species (*n* = 10), as well as from canines harboring other filarial diseases, such as *A. reconditum* (*n* = 2), *Brugia patei* (*n* = 1), *Onchocerca lupi* (*n* = 1). These samples were obtained from Slovakia (DR/DI; AR) and Romania (BP; OL).

All reactions performed to validate sensitivity and specificity were carried out in triplicates under the conditions described in the manuscript.

### Study design and statistical evaluation

The prevalence rates for canine and feline hosts were calculated based on the output of molecular testing. Animals were subsequently stratified into various subgroups based on demographic and physiological parameters such as sex, age, and weight for in-depth analysis. A subset of animals, comprising 16 dogs and 12 cats, were excluded from detailed analysis due to incomplete data, though they were incorporated in the overall prevalence estimation (Additional File1: Table S1). Additionally, only animals aged 7 months or older were considered for the prevalence analysis. Statistical assessments were conducted using GraphPad Prism version 8.0 (GraphPad Software, La Jolla, CA, USA), with chi-square test applied to discern significant differences among groups. A p-value less than 0.05 was earmarked as the threshold for statistical significance.

## Results

### Prevalence of parasitic infections

**Fig. 2:**
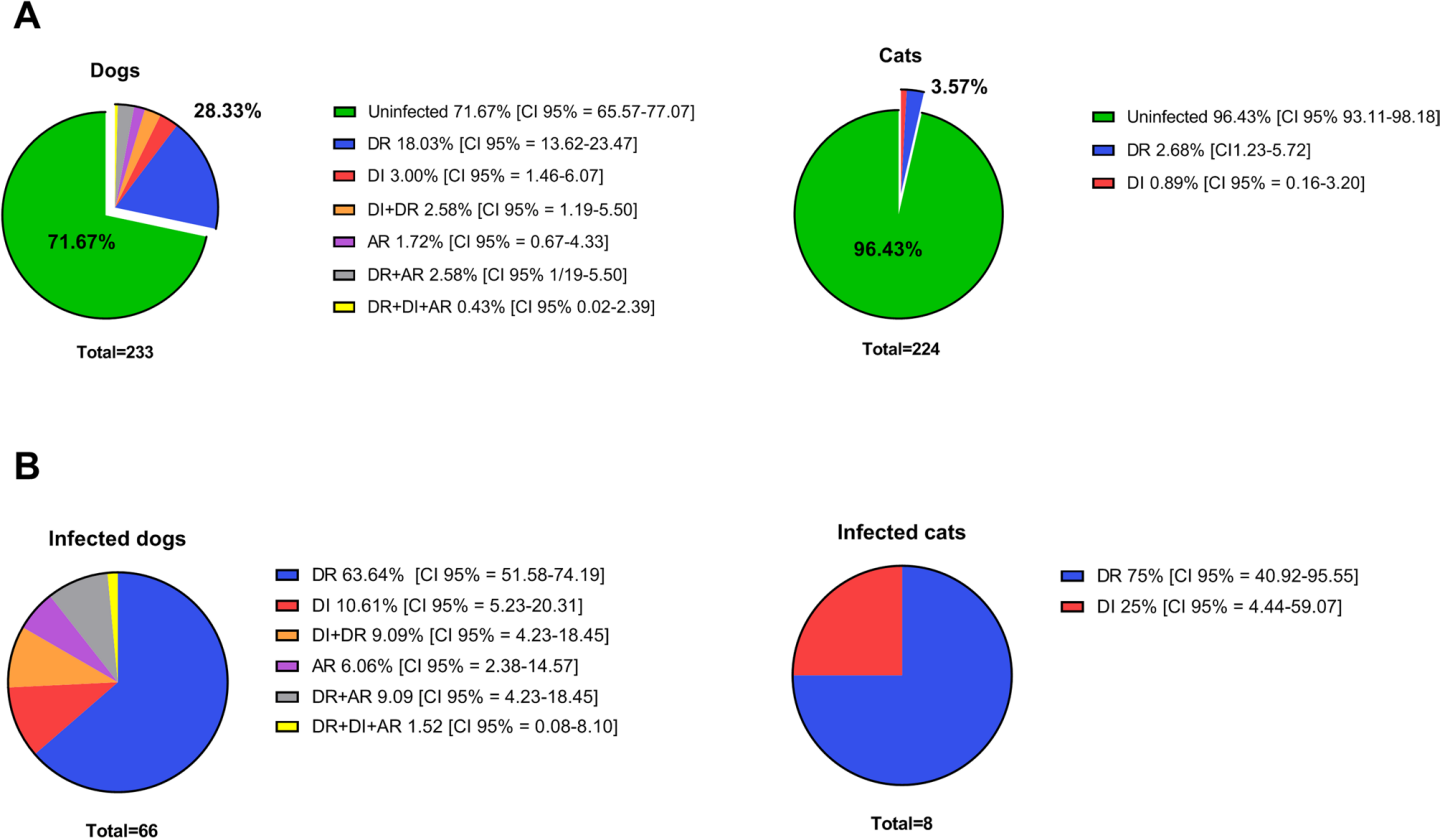
Prevalence of *Dirofilaria* and *Acanthocheilonema* infections in dogs and cats. (**A**) Occurrence frequency of each species, including both mono- and co-infections, in dogs and cats compared to negative (uninfected) animals. (**B**) Detailed analysis of infection patterns among infected dogs and cats. DR - *D. repens*; DI - *D. immitis*; AR - *A. reconditum*

Molecular examination revealed the presence of *D. repens*, *D. immitis*, or *A. reconditum* in 66 out of 233 canine samples, yielding in an overall prevalence of 28.3%, and in 8 out of 224 feline samples, yielding a prevalence of 3.6% (Fig. 2).

The geographic distribution of these infections spanned all areas under study (Fig. 1), with various infection dynamics observed, including single infections (*D. repens*, *D. immitis*, or *A. reconditum*) and dual parasitic co-infections (*D. repens* + *D. immitis* or *D. repens* + *A. reconditum*). Notably, a single case involved a triple infection in a dog, encompassing *D. repens*, *D. immitis*, and *A. reconditum*. Predominantly *D. repens* was the most prevalent parasite among both dogs and cats across all areas, with *A. reconditum* being the least encountered (Fig. 2).

### Regional differences in prevalence

**Fig. 3:**
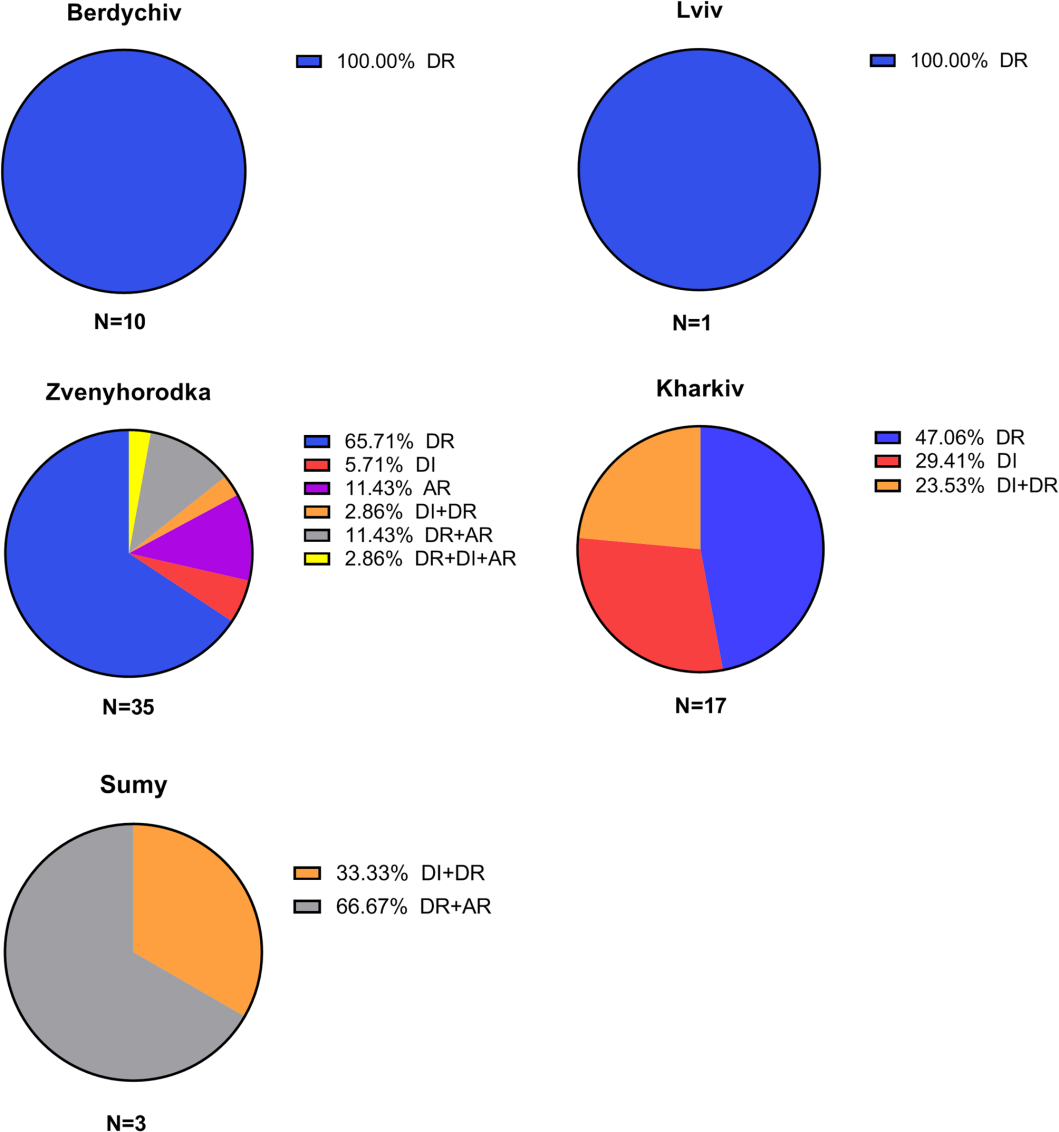
Infection patterns frequency in affected dogs across Berdychiv, Lviv, Kharkiv, Sumy and areas. DR - *D. repens*; DI - *D. immitis*; AR - *A. reconditum*

Regional prevalence was calculated by including all filarial species. Zvenyhorodka recorded a canine prevalence of 50.0% [95% CI = 38.59–61.40] (Fig. 1) and showcased the broadest spectrum of infection types, with each pattern of infection represented (Fig. 3). In Lviv and Berdychiv, dogs were solely infected with *D. repens*, with prevalences of 9.1% [95% CI = 0.46–37.73] and 34.4% [95% CI = 19.94–52.65], respectively. Kharkiv and Sumy had comparable (χ2 = 0,027; df = 1; *p* = 0,867) prevalence, 16.0% [CI 95% = 10.26–24.19] and 17.7% [CI 95% = 6.19–41.02] (Fig. 1), with co-infections occurring exclusively in Sumy (Fig. 2).

In cats, prevalence was similar (χ2 = 0.113; df = 1; *p* = 0.736) across areas: 3.9% [CI 95% = 1.69–8.89] in Kharkiv and 3.1% [CI 95% = 0.84–8.70] in Sumy (Fig. 1).

Detailed information on the regional prevalence in dogs and cats across the examined areas is provided in Fig. 3 and Additional File 1: Table S2. Complete statistical results regarding regional differences in prevalence among dogs and cats are presented in Additional File 1: Table S3.

### Risk factors for infection prevalence

**Fig. 4:**
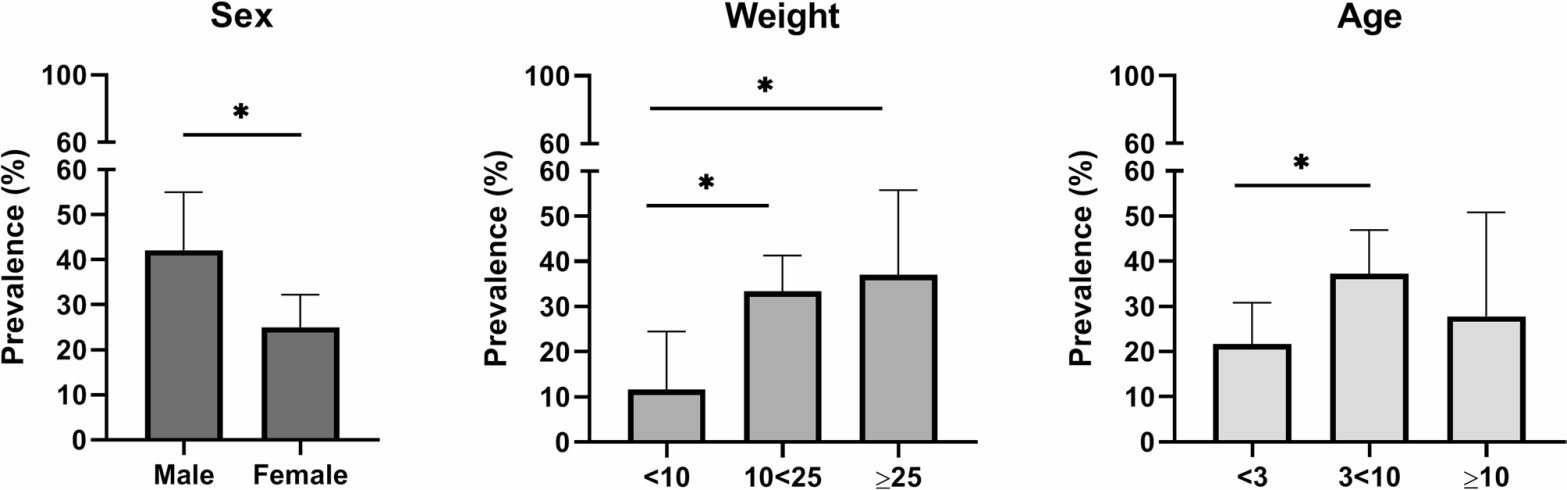
Comparative prevalence of filarial infections across dogs categorized by sex, age and weight. All dogs in the study were grouped according to their sex (male/female), weight (< 10 kg; 10–25 kg; ≥25 kg), and age (< 3 years; 3–10 years; ≥10 years)

Risk factors for infection were assessed based on the overall prevalence of all filarial species across the defined groups. Prevalence was significantly higher (χ2 = 5,913; df = 1; *p* = 0.015) in males (42.1% [CI 95% = 30.19–50.02]) compared to females (25.4% [CI 95% = 18.93–32.24]). Regarding weight, the lowest prevalence was observed in the lightest dogs (< 10 kg) at 11.6% [95% CI: 5.07–24.48], compared to 33.3% [95% CI: 26.22–41.29] in dogs weighing 10–25 kg (χ² = 7.704; df = 1; *p* = 0.006) and 37.0% [95% CI: 21.53–55.77] in those over 25 kg (χ² = 6.360; df = 1; *p* = 0.012). Age also influenced prevalence, with dogs aged 3–10 years being significantly more likely (χ2 = 5.805; df = 1; *p* = 0.016) to be infected (37.3% [CI 95% = 28.49–49.94]) compared to those younger than 3 years (21.6% [CI 95% = 14.62–30.84]). In the oldest age group (> 10 years), the prevalence was 27.8% [95% CI: 12.5–50.87]; however, no significant differences were observed when compared to group of younger dogs (Fig. 4). The complete statistical results related to risk factors in dogs are presented in Additional File 1: Table S4. Of eight infected cats, 75% harbored *D. repens* and 25% had *D. immitis* (Fig. 2), with cases ranging from 10 months to 10 years old, predominantly in females (75%).

### Assessment of qPCR analytical sensitivity and primers specificity

**Fig. 5:**
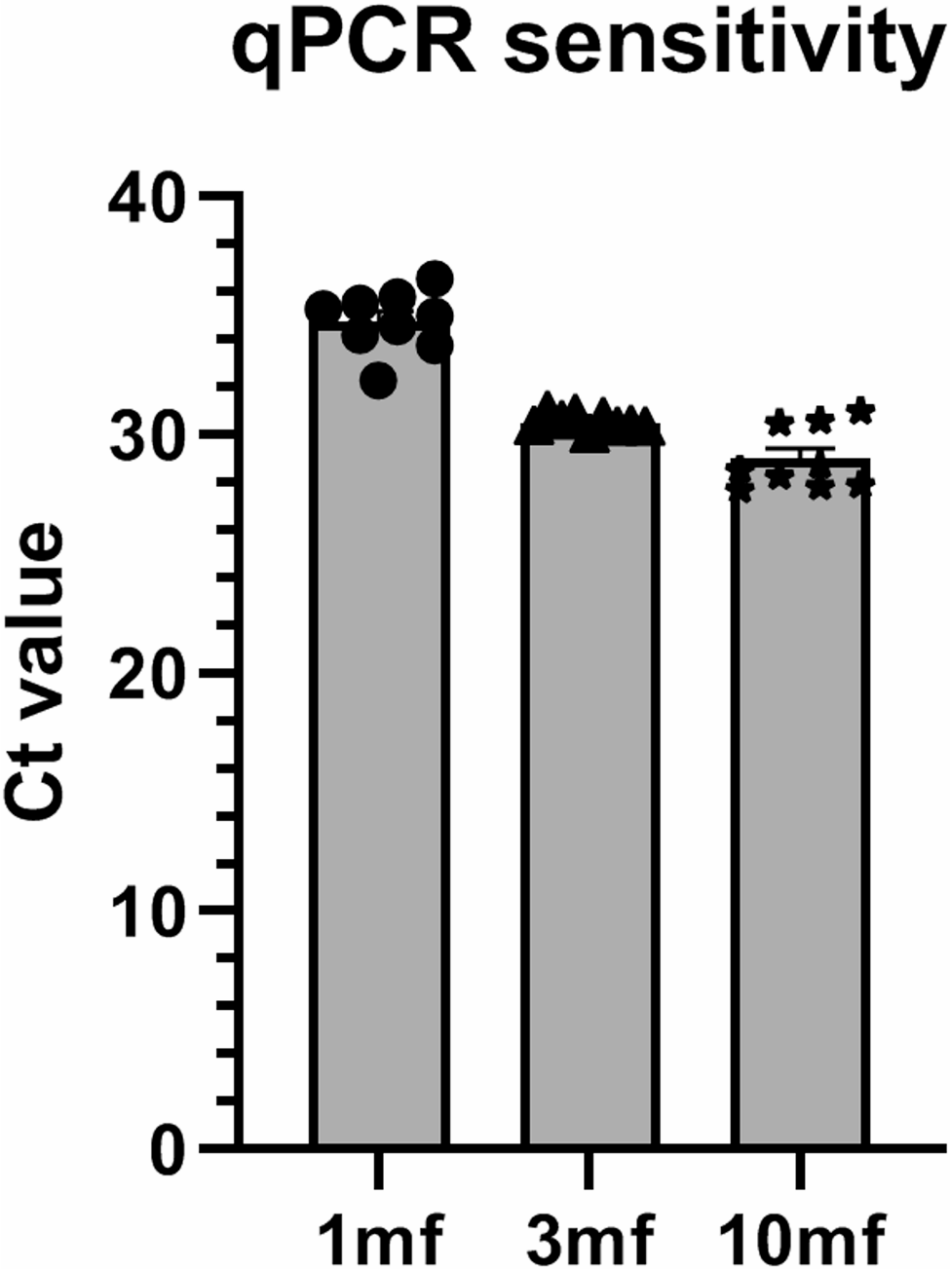
Sensitivity evaluation of the Real-Time assay using pan-filarial primers targeting*cox1*. The assay was tested with gDNA from 1, 3, or 10 microfilariae (mf). Reactions were performed in triplicate across three biological replicates using blood from three different dogs

The analytical sensitivity of the assay was determined to be 1 mf per 300 µL of blood used for DNA isolation (Fig. 5).

**Fig. 6:**
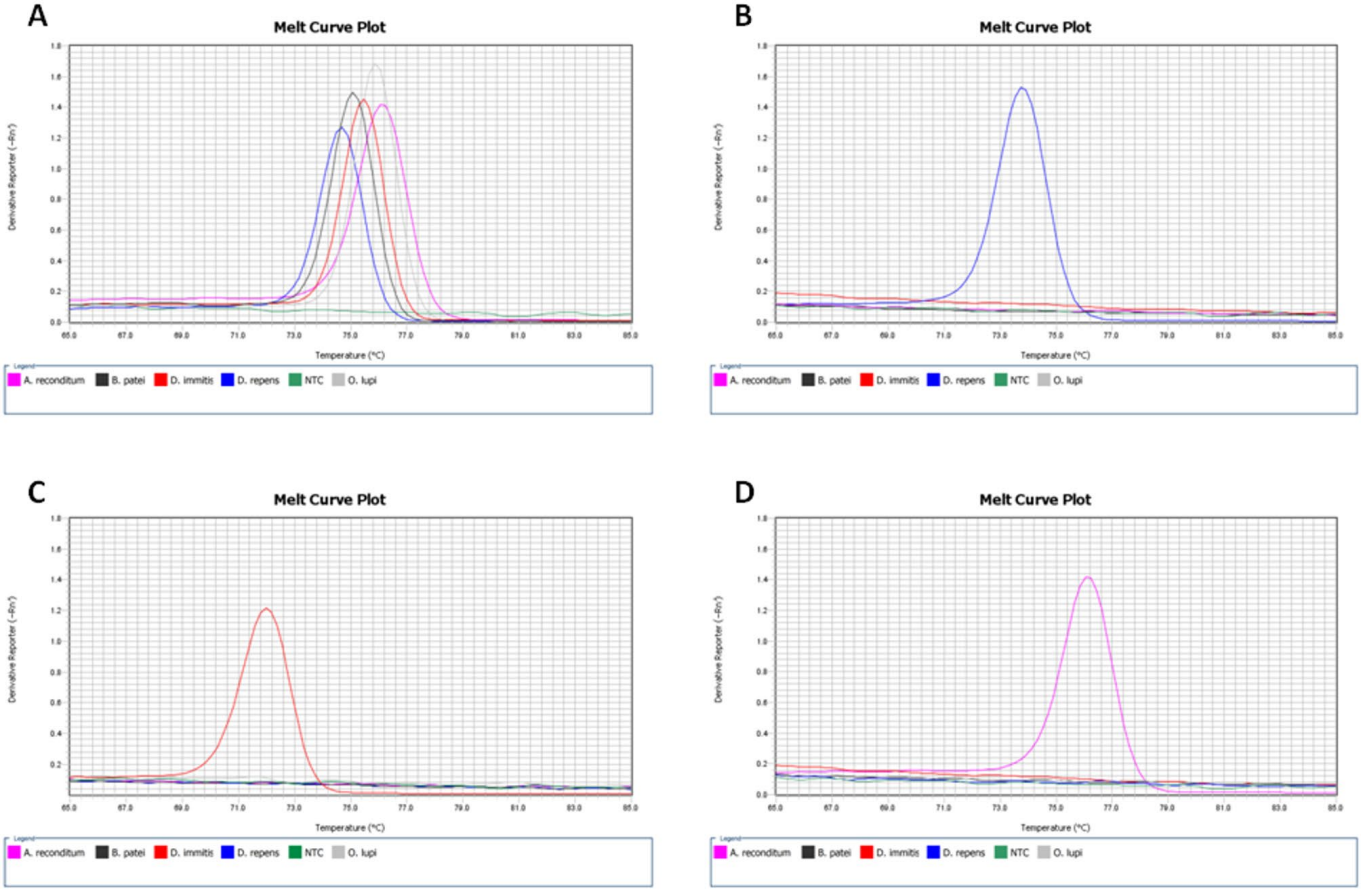
Melt curve analysis of the products amplified using gene-specific primers. Plots display melt curves corresponding to primers specific for Onchocercidae family (**A**), *D. repens* (**B**), *D. immitis* (**C**), and *A. reconditum* (**D**). Primers specificity was assessed with genomic DNA isolated from blood from dogs infected with *D. repens* (blue), *D. immitis* (red) and *A. reconditum* (purple), *B. patei* (black), *O. lupi* (grey). NTC (green) implies „no target control”

The pan-filarial primers were specific to each species within the Onchocercidae family examined. No cross-priming was observed when using species-specific primers (Fig. 6).

Notably, in silico analysis revealed primers targeting *D. repens* may cross prime with *Dirofilaria* sp. ‘hongkongensis’ cautioning against their use in the endemic areas (Additional File 1: Fig. S2).

## Discussion

This study presents a detailed molecular investigation into the prevalence of dirofilariosis among stray canine and feline populations in Ukraine. Through our research, we have not only corroborated the endemic presence of *D. repens* and *D. immitis* within Ukraine, but have also, somewhat unexpectedly, identified several cases of *A. reconditum*. This marks the inaugural report of this particular vector-borne filaria within the area.

Our findings indicate an overall filariosis prevalence of 28.3% among dogs from five distinct area. Specifically, *D. repens* infections were observed in 18% of dogs as monoinfections and in 23.6% of dogs when considering co-infections with other species. Conversely, *D. immitis* was found to account for 3% of monoinfections and 6% when the co-infections where included. These results appear to align with other studies investigating dirofilariosis within the canine population of Ukraine. For instance, Bajer et al. reported that 18.9% and 3.8% of dogs relocated from Ukraine to Poland amid the ongoing military conflict were found to be infected with *D. repens* and *D. immitis*, respectively. Moreover, two dogs were identified as having co-infections with both species [10].

The focus of dirofilariosis research within Ukraine has predominantly been the Kharkiv area, where notable presence of both *D. repens* and *D. immitis* has been identified within the canine population. Over a decade spanning 2009 to 2019, a considerable portion (37%) of dogs were found to be infected. The prevalence of *D. repens* infections notably overshadows those of *D. immitis*, comprising 97.6 ± 0.2% of infections compared to 2.9 ± 0.5% for *D. immitis* [22]. A subsequent study conducted over 2018 and 2019 revealed infection in 24 out of 120 dogs, equating to a 21.4% infection rate, with a distribution of 14 cases of *D. immitis* and 10 of *D. repens* [23]. Contrastingly, our investigation indicates a lower total prevalence of 16.03% in this area. Among the infected dogs, *D. repens* was more prevalent (47.1%) compared to *D. immitis* (29.4%), with co-infections of both species observed in 23.5% of the affected dogs. Additionally, during the winter hunting season of 2019/2020, post-mortem examinations on 27 red foxes from the area were performed. This examination discovered that six foxes harbored adult *D. immitis* worms within the right heart ventricle, pulmonary trunk, and pulmonary arteries [24]. Xenomonitoring in this area also demonstrated that mosquitoes were found to be infected with *Dirofilaria* spp. in 11 districts within Kharkiv area and the city itself, with a prevalence of 4.5 ± 0.2% [22].

In another area explored in our study, Sumy, we noted an overall prevalence of 17.7%. In particular, two dogs were found to be co-infected with *D. repens* and *D. immitis*, and one dog with *D. repens* and *A. reconditum*. It’s worth mentioning that the sample size in this area was the smallest (*n* = 17) compared to all other areas studied. A separate study conducted in this area between 2010 and 2018 reported a 9.4% prevalence of *Dirofilaria* spp., with *D. repens* found in 94.8% of the infected dogs [25]. Additionally, the prevalence of *Dirofilaria* spp. in mosquitoes, based on microscopic examination, was estimated to be 0.4% in this area [26].

Further areas considered in our analysis included Berdychiv and Zvenyhorodka, where markedly higher prevalence rates were observed (34.5% and 50%, respectively) compared to the eastern areas (Sumy, Kharkiv). Notably, despite the relatively minor geographical distance between these two cities, the patterns of infection exhibited substantial variation. In Berdychiv, all cases of infection were attributed to *D. repens*, whereas in Zvenyohorodka, a wide array of infection patterns was noted, including monoinfections of each species, co-infections (*D. repens* + *D. immitis*, *D. repens* + *A. reconditum*), and even a case of triple infection. Regrettably, to our knowledge, canine dirofilariosis has not previously been studied in these areas, based on the available literature in both Ukrainian and English. The only related data we could retrieve was from a 2016 study in Zhytomyr that focused on hematological changes in dogs with dirofilariosis, specifically targeting hunting breeds, which included 10 infected dogs, though the species were not distinguished [27].

Adjacent to Zhytomyr Region (Berdychiv) and Cherkasy Region (Zvenyhorodka) is Kyiv, situated approximately 150 km away from each city in a direct line. In Kyiv, of 23 samples collected in 2011, 2 (9%) and 1 (4%) tested positive for *D. repens* and *D. immitis*, respectively [11]. In Bila Tserkva, the largest city in the Kyiv Region, 9% of dogs were found to be infected with *Dirofilaria*, although the data did not specify the species [28]. Alsarraf et al. reported that among 155 samples collected between 2017 and 2019 from Kamianets-Podilskyi (adjacent to Zhytomyr Region) and Vinnytsia (bordering both Cherkasy and Zhytomyr Regions), 3.8% of pet dogs were infected with *D. repens* [12].

Salamatin et al. have documented human dirofilariosis across all regions in Ukraine over the years. Until 2012, within the areas considered in our study, incidence rates varied, with Lviv and Zhytomyr showing the lowest incidence, ranging from 0.07 to 1.68 per 100,000 people. In contrast, the incidence recorded in Cherkasy and Kharkiv regions ranged from 2.43 to 3.71, with the highest frequencies recorded in Sumy, ranging from 4.90 to 5.45/100,000 [13].

In our study we also diagnosed acanthocheilonemiosis in several dogs within Ukraine, highlighting the emergence of *A. reconditum* (previously known as *Dipetalonema reconditum*) as a filarial parasite of domestic and wild canids. The parasite is transmitted by arthropods such as fleas, lice and ticks. Although *A. reconditum* infections are typically considered non-pathogenic, they can lead to clinical manifestations such as anemia, skin issues (e.g., abrasions and pruritus), tissue-related pathologies, cachexia, and respiratory distress [29, 30]. Furthermore, some hematological (e.g., leukocytosis, eosinophilia, monocytosis) and biochemical abnormalities (e.g., increased total serum proteins, albumins, and globulins) may be observed during infection [30]. Despite its traditionally low clinical significance, the increasing prevalence of *A. reconditum* in canine populations in specific areas [29, 31] and the identification of a few cases of human acanthocheilonemiosis in recent years [32, 33] underline the public health importance of this filaria. *Acanthocheilonema reconditum* is present in a wide range of geographical areas globally, including the Mediterranean Basin, the Middle East, South Africa, South America, and Oceania [32]. In our study, *A. reconditum* infections were identified in two Ukrainian areas (Sumy and Zvenyohorodka), with an overall prevalence of 4.73% among the examined dogs, including co-infections with *D. repens*. Notably, while a single case of triple infection was observed, no co-infections with *D. immitis* alone were detected.

Moreover, this study delves into feline dirofilariosis within Ukraine. Prior to this research, only a single case of dirofilariosis in cats had been documented in Ukraine, where adult worms identified as *D. repens* were surgically removed from the scrotum and spermatic cord in Kiev in 2003 [34]. Feline dirofilariosis, although less common than in canines, has been reported globally [35]. In our study, the overall prevalence of *Dirofilaria* in cats was determined to be 3.6%, with 2.7% attributed to *D. repens* and 0.9% to *D. immitis*. These findings are generally consistent with data from other countries, where infection frequencies vary from less than 1% to several percent, depending on the areas and diagnostic methods employed [35]. However, given that our diagnostic approach relied solely on qPCR, which is dependent on detecting DNA in the bloodstream, it is plausible that the overall prevalence in our study may be underestimated.

Cats are often considered less susceptible hosts for *Dirofilaria* compared to dogs, with approximately 25% of cats naturally resistant to *D. immitis* infection [36]. In cats, only small proportion of L3 larvae progress to the adult stage [37]. Although cases of active microfilaremia have been described for *Dirofilaria* spp [38, 39]., only about 20% of infected cats exhibit microfilaremia, which is typically short-lived and characterized by a low microfilariae load [36, 39]. This presents challenges for accurate diagnosis. Serological tests, commonly used to detect feline heartworm, are often considered the gold standard, particularly for occult and subclinical infections. However, the prolonged persistence of *Dirofilaria* specific antibody titers or antigenemia post-infection can result in false-positive outcomes [36]. Currently, there are no commercial tests available for *D. repens*. A limited number of studies have explored cell-free DNA (cfDNA) as a diagnostic marker for canine dirofilariosis [19, 40], which could be particularly valuable in occult infections. Unfortunately, research on *Dirofilaria*-derived cfDNA in cats remains scarce.

None of the cats in our study were found to be infected with *A. reconditum*. While the infective stage of *A. reconditum* can develop in the feline flea *Ctenocephalides felis felis*, infections in cats are exceedingly rare, with isolated cases reported only in Thailand [41].

In addition to the primary focus of our study, we also examined risk factors for infections in dogs, such as host sex, age, and size (weight). Helminth infections are known to be influenced by the host’s sex, with males typically experiencing more intense and prevalent infections compared to females. This disparity is attributed to ecological, behavioral, and physiological differences between the sexes, including interactions among sex hormones, chromosomes, the microbiome, and the immune system [42]. In our study, each of the filaria species detected occurred significantly more frequently in males than in females, a pattern that has been observed in several studies on *Dirofilaria* and *Acanthocheilonema* [12, 23, 29, 43, 44].

Few reports have indicated that the prevalence of filariasis is lowest in dogs under 3 years of age compared to older animals [12, 23, 43, 44], which aligns with our findings − 21.6% in dogs under 3 years, compared to 37.3% in those aged 3–10 years and 27.8% in dogs over 10 years old. This trend is likely due to the extended prepatent period before microfilariae become detectable and the increased exposure to mosquito bites experienced by older animals.

Our study, in line with a few others, also demonstrated that a dog’s size significantly influences the frequency of infection [23, 45]. Although the exact reason for this association remains uncertain, it may be related to behavioral differences in larger dogs, who may explore a wider range of environments and provide larger surface area for contact with mosquitoes.

In conclusion, our comprehensive analysis of stray dogs and cats across four Ukrainian cities highlights the notable prevalence of *Dirofilaria* spp., reaffirming the endemic nature of these parasites in the areas. The detection of *A. reconditum* infections introduces a new dimension to the vector-borne disease landscape in Ukraine, emphasizing the complexity of these diseases within the country. Pet owners, veterinarians, and physicians need to maintain heightened awareness to ensure accurate diagnosis of filariasis and the timely implementation of therapeutic and preventive treatments in companion animals.

## Electronic supplementary material

Below is the link to the electronic supplementary material.

Supplementary Material 1

## Data Availability

All data generated or analyzed during this study are included in this published article and its Supplementary Information files. Raw data and sequencing information are available in the Dane Badawcze UW repository (10.58132/67HY9Z).
