# Supplementary material for: Molecular investigation of Dirofilaria repens, Dirofilaria immitis and Acanthocheilonema reconditum in stray dogs and cats in Ukraine
Source: BMC Vet Res. 2025 Jul 5;21:438. doi: 10.1186/s12917-025-04867-w (PMC12228257; doi:10.1186/s12917-025-04867-w)
Supplement: Supplementary file 1 — Supplementary Material 1 [file 12917_2025_4867_MOESM1_ESM.pdf]

Supplementary information

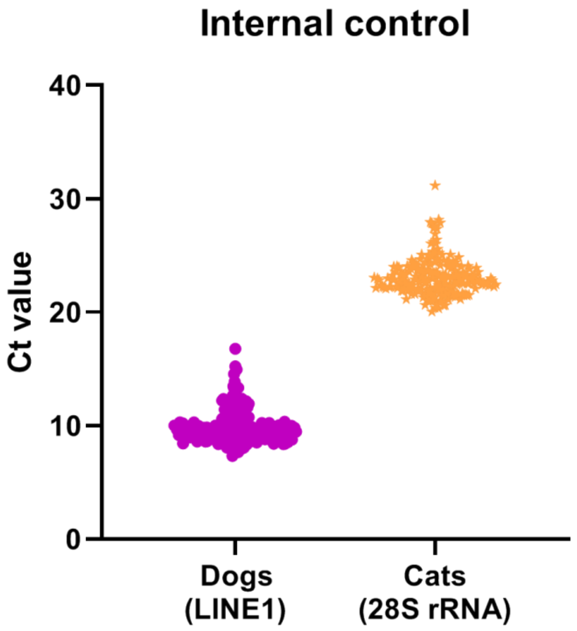

Fig. S1 Comparison of Ct values for internal extraction/amplification controls in Real-Time PCR, using primers targeting LINE1 for dogs and 28S rRNA for cats.

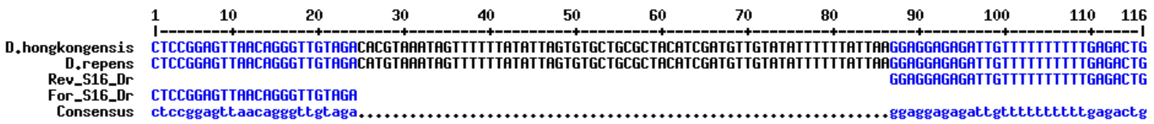

Fig. S2 The specificity of the primers targeted to a fragment of the *Dirofilaria repens 16S rRNA* gene was aligned with homologous sequences from *Dirofilaria* sp. 'hongkongensis'. The GenBank accession numbers of the sequences used in the analysis are: *D. repens*: NC_029975.1; *Dirofilaria* sp. 'hongkongensis': NC_031365.1.

.

Table S1 Detailed distribution of canine and feline blood samples across regions, categorized by sex, age, and weight*

*NA, non-analyzed

Origin	Dogs/cats	Sex	Age	Weight	Total number
Lviv	Dogs	Males: 5<br>Females: 3	<3 years: 1<br>3-10 years: 4<br>≥10 years: 3	<10: 2<br>10-25: 2<br>≥25: 4	11
		Unknown: 3			
Berdychiv	Dogs	Males: 9<br>Females: 20	<3 years: 11<br>3-10 years: 16<br>≥10 years: 2	<10: 0<br>10-25: 21<br>≥25: 8	29
Zvenyhorodka	Dogs	Males: 22<br>Females: 48	<3 years: 32<br>3-10 years: 33<br>≥10 years: 5	<10: 3<br>10-25: 64<br>≥25: 3	70
Sumy	Dogs	Males: 5<br>Females: 12	<3 years: 0<br>3-10 years: 11<br>≥10 years: 6	<10: 5<br>10-25: 8<br>≥25: 4	17
	Cats	Males: 17<br>Females: 80	<3 years: 52<br>3-10 years: 37<br>≥10 years: 7	NA	97
		Unknown: 1			
Kharkiv	Dogs	Males: 16<br>Females: 77	<3 years: 53<br>3-10 years: 38<br>≥10 years: 2	<10: 33<br>10-25: 52<br>≥25: 8	106
		Unknown: 13			
	Cats	Males: 26<br>Females: 90	<3 years: 79<br>3-10 years: 37<br>≥10 years: 0	NA	127
		Unknown: 11			

Table S2 Detailed distribution of infection patterns in dogs and cats across examined regions*

*DR, *Dirofilaria repens*; DI, *Dirofilaria immitis*; AR, *Acanthocheilonema reconditum*.

Origin	Dogs/cats	DR	DI	AR	DR+DI	DR+AR	DI+AR	DR+DI+AR	Total number
Lviv	Dogs	1	-	-	-	-	-	-	1
Berdychiv	Dogs	10	-	-	-	-	-	-	10
Zvenyhorodka	Dogs	23	2	4	1	4	-	1	35
Sumy	Dogs	-	-	-	1	2	-	-	3
	Cats	3		-	-	-	-	-	3
Kharkiv	Dogs	8	5	-	4	-	-	-	17
	Cats	3	2	-	-	-	-	-	5

Table S3 Chi-square test results for differences in infection prevalence by region, sex, weight, and age in dogs*

* $\chi2$ - Chi-square; df - degrees of freedom; *p* - statistical significance

**Berdychiv**					
**Lviv**	$\chi2 = 2.579$<br>df = 1<br>$p = 0.108$				
**Kharkiv**	$\chi2 = 4.842$<br>df =1<br>$p = 0.027$	$\chi2 = 0.369$<br>df = 1<br>$p = 0.543$			
**Sumy**	$\chi2 = 2.498$<br>df =1<br>$p = 0.027$	$\chi2 = 0.399$<br>df = 1<br>$p = 0.5275$	$\chi2 = 0.027$<br>df =1<br>$p = 0.867$		
**Zvenyhorodka**	$\chi2 = 1.991$<br>df =1<br>$p = 0.158$	$\chi2 = 6.443$<br>df = 1<br>$p = 0.011$	$\chi2 = 23.360$<br>df =1<br>$p = 0.0001$	$\chi2 = 5.820$<br>df =1<br>$p = 0.015$	
	**Berdychiv**	**Lviv**	**Kharkiv**	**Sumy**	**Zvenyhorodka**

Table S4 Chi-square test results for regional differences in infection prevalence*

* $\chi 2$ - Chi-square; df - degrees of freedom; *p* - statistical significance

	**$\chi 2$**	**df**	***p***
**Males vs females**	5.913	1	0.015
**<10 kg vs 10–25 kg**	7.704	1	0.006
**<10 kg vs ≥25 kg**	6.360	1	0.012
**10–25 kg vs ≥25 kg**	0.14	1	0.709
**<3 years vs 3-10 years**	5.805	1	0.016
**<3 years vs ≥10 years**	0.326	1	0.568
**<3 years vs 3-10 years**	0.598	1	0.440
